# Could Sodium Chloride be an Environmental Trigger for Immune-Mediated Diseases? An Overview of the Experimental and Clinical Evidence

**DOI:** 10.3389/fphys.2018.00440

**Published:** 2018-04-24

**Authors:** Eric Toussirot, Matthieu Béreau, Charline Vauchy, Philippe Saas

**Affiliations:** ^1^Institut National de la Santé et de la Recherche Médicale CIC-1431, Centre d'Investigation Clinique Biothérapie, University Hospital Besançon, Besançon, France; ^2^Fédération Hospitalo-Universitaire INCREASE, University Hospital Besançon, Besançon, France; ^3^Rhumatologie, University Hospital Besançon, Besançon, France; ^4^Département de Thérapeutique et EPILAB EA4266: “Epigénétique des Infections Virales et des Maladies Inflammatoires”, Université Bourgogne Franche-Comté, Besançon, France; ^5^Department of Neurology, University Hospital Besançon, Besançon, France; ^6^Institut National de la Santé et de la Recherche Médicale U1098, Etablissement Français du Sang Bourgogne Franche-Comté, Université Bourgogne Franche-Comté, Interactions Hôte-Greffon-Tumeurs, LabEx LipSTIC, Besançon, France

**Keywords:** autoimmune diseases, sodium chloride, IL-23, Th17, SGK1

## Abstract

Immune mediated diseases (IMDs) are complex chronic inflammatory diseases involving genetic and environmental factors. Salt intake has been proposed as a diet factor that can influence the immune response. Indeed, experimental data report the influence of sodium chloride on the differentiation of naive CD4^+^ T cells into IL-17 secreting T helper (Th) cells (Th17 cells), by a mechanism involving the serum glucocorticoid kinase-1 (SGK1) that promotes the expression of the IL-23 receptor (IL-23R). The IL-23/IL-23R is critical for pathogenic inflammatory Th17 cell differentiation. Experimental data in murine models of arthritis, colitis and encephalomyelitis corroborate these findings. This manuscript reviews the current knowledge on the effects of sodium chloride on innate and adaptive immunity. We also performed a systematic literature review for clinical studies examining the relationships between salt consumption and the development or the activity/severity of the most common IMDs mediated by the IL-23/Th17 pathway, i.e., rheumatoid arthritis (RA), multiple sclerosis (MS), and Crohn's disease (CD). Nine studies were found, 4 in RA, 4 in MS and 1 in CD. An association was found between developments of anti-citrullinated protein antibody (ACPA) positive RA in smokers and salt intake, but these results were not confirmed in another study. For MS, no association was observed in pediatric subjects while in adult patients, a link was found between salt intake and disease activity. However, this result was not confirmed in another study. These conflicting results highlight the fact that further evaluation in human IMDs is required. Moreover, physicians need to develop clinical trials with diet interventions to evaluate the impact of low salt intake on disease activity/severity of IMDs.

## Introduction

Immune-mediated diseases (IMDs) are complex chronic inflammatory disorders involving the contribution of different predisposing factors. Indeed, both specific genetic backgrounds and environmental factors participate in their pathogenesis. Environmental factors have been implicated according to the prevalence of these diseases in certain countries or geographical areas, and/or through exposure of the population to local factors, but lifestyle habits as well as diet are also involved. The most common IMDs are type I diabetes, systemic lupus erythematosus (SLE), rheumatoid arthritis (RA), psoriasis, multiple sclerosis (MS), and inflammatory bowel diseases, such as Crohn's disease (CD). Contributing environmental factors are specific for each IMD, and for instance, smoking is a well identified factor for the development of RA or CD, while ultraviolet exposure is involved in SLE. The influence of diet has long been suspected as a contributing pathogenic factor for IMD. For instance, a Mediterranean diet is thought to influence the occurrence of RA (Casas et al., 2017). Diet is also a modifiable environmental risk factor, and thus, the identification of a specific eating habit or food component in IMDs could help physicians in the prevention and treatment of these diseases. However, in general, the influence of diet on the pathogenesis of most IMDs is currently unknown.

It has recently been reported that salt in the diet could influence the development of certain IMDs, such as arthritis, CD, or MS (Croxford et al., 2013; van der Meer and Netea, 2013; Sigaux et al., 2017). Sodium is an extracellular cation that plays a fundamental role in the physiology of humans. Indeed, sodium is the most abundant cation in the organism, and is involved in osmotic balance, extracellular fluid volume as well as membrane potential of cells. Most dietary sodium is consumed as common salt i.e., sodium chloride. It is present in common food and is added when cooking or in food preparation. A high-salt diet has been implicated in certain pathological conditions, such as hypertension, cardiovascular and kidney diseases. It is well known that the mean daily salt intake is excessive in most western countries (between 7 and 18 g) and even higher in Asia, and in excess compared to the World Health Organization recommendations (<5 g daily; Brown et al., 2009). Recent data have highlighted the potential role of high-salt diet in the development of experimental IMD models, such as collagen-induced arthritis (CIA), experimental autoimmune encephalomyelitis (EAE), and experimental colitis. Indeed, mice receiving a high-salt diet have been shown to experience exacerbated colitis, CIA or EAE (Table 1). This has been associated with either an increase of inflammatory immune responses (e.g., M1 macrophages; Ip and Medzhitov, 2015; Hucke et al., 2016), pathogenic T cells (Kleinewietfeld et al., 2013; Wu et al., 2013, 2018; Wei et al., 2017; Aguiar et al., 2018), or autoantibodies (Sehnert et al., 2014) or a decrease of protective immune functions (e.g., regulatory CD4^+^ T cell [Treg]; Hernandez et al., 2015; Wu et al., 2018), and IL-4/IL-13 activated M2 macrophages suppressive activity (Binger et al., 2015a). These findings suggest that high-salt diet may affect the functions of innate (i.e., macrophages; Binger et al., 2015a; Hucke et al., 2016) or innate lymphoid cells [ILC] ILC3 (Aguiar et al., 2018) and adaptative (i.e., CD4^+^ T cells) immune cells. For instance, sodium chloride favored the polarization of pathogenic CD4^+^ T cells toward a T helper 17 (Th17) phenotype (see below and Table 1). All these data are supported by studies performed in healthy volunteers showing that a controlled high-salt diet is correlated with an increase of circulating inflammatory monocytes counts, as well as pro-inflammatory cytokines (e.g., IL-6, IL-17, or IL-23) (Zhou et al., 2013; Yi et al., 2015). Recently, circulating Th17 cells were analyzed in an exploratory pilot cohort of 8 healthy male volunteers before and after a high-salt challenge (Yi et al., 2015). A significant increase of IL-17A^+^ TNF^+^ CD4^+^ T cells was observed after this high-salt challenge. Altogether, this sheds new insights on the relationship between environmental factors, diet, and specific IMDs.

**Table 1:** Effects of high-salt diet on experimental immune-mediated diseases.

**Author (Reference)**	**Experimental disease**	**Experimental model**	**Effects on the disease**	**Immune mechanisms**
Sehnert (Ip and Medzhitov, 2015)	Arthritis	CIA	Increase incidence of arthritis in high-salt group with increased infiltration of inflammatory cells in the joint associated with more pronounced cartilage and bone destruction	Increased levels of pathogenic IgG2 anti-bovine collagen II auto-antibodies
Jung (Jung et al., 2014)	Arthritis	CIA	More severe joint inflammation in the high-salt diet fed mice	Not directly tested. However, an increase of RORγT in T cells from high-salt diet fed mice and an enhanced capacity to differentiate into Th17 cells
Wei (Wei et al., 2017)	Colitis	TNBS-induced colitis	More severe TNBS-induced colitis in high-salt diet fed mice (inflammatory scores and colon weight)	Increased Th17 response in TNBS-induced colitis of high-salt diet fed mice, attested by increased frequency of IL-17A^+^ lamina propria CD4^+^ T cells and increased levels of IL-6, IL-17A and IL-21 in colonic tissues
Hernandez (Hernandez et al., 2015)	Colitis	ATIC	Mouse Treg exposed to high-salt are less efficient than control Treg to prevent naive CD4^+^ T cell-induced colitis as assessed by weight loss	High-salt conditions induces a Th1 phenotype of activated Treg
Monteleone (Monteleone et al., 2017)	Colitis	TNBS- and DSS-induced colitis	More severe TNBS-induced colitis in high-salt diet fed mice (histologic scores) and correction by a p38 kinase inhibitor treatment More severe DSS-induced colitis in high-salt diet fed mice (histologic scores)	Increased mRNA levels of *Rorgt* and *Il17a* in high-salt diet fed mice in the TNBS model
Aguiar (Aguiar et al., 2018)	Colitis	TNBS- and DSS-induced colitis	More severe DSS- and TNBS-induced colitis in high-salt diet fed mice (clinical and histological scores in both models; reduced colon length and decreased mouse survival in DSS- and TNBS-induced colitis, respectively)	Increased frequency of RORγT^+^ CD4^+^ T cells from the lamina propria of high-salt diet fed mice
Hucke (Hucke et al., 2016)	EAE	MOG_35−55_ immunization in C57BL/6 mice	A more severe clinical score in high salt fed mice associated with increased myeloid cell infiltrate in the white matter	Macrophage skewing through a pro-inflammatory M1 phenotype (enhanced iNOS and costimulatory molecule expression as well as highly responsive to *in vitro* LPS stimulation)
Wu (Wu et al., 2013)	EAE	MOG_35−55_ immunization in C57BL/6 mice	A more severe clinical score in high salt fed mice that is reduced in *Sgk1*-deficient mice	Increased frequency of IL-17^+^ CD4^+^ T cells in the CNS and mLN of high-salt diet fed mice
Kleinewietfeld (Kleinewietfeld et al., 2013)	EAE	MOG_35−55_ immunization in C57BL/6 mice	A more severe clinical score in high salt fed mice associated with increased myeloid and T cell infiltrate in the spinal cord	Increased mRNA levels of *Il17a* and *Rorc* in the spinal cord of high-salt diet fed mice Increased mRNA levels of *Il17a, Nfat5*, and *Sgk1* in splenocytes of high-salt diet fed mice Enhanced Th17 polarization of splenic T cells from high-salt diet fed mice experiencing EAE in response to MOG peptide re-stimulation

*ATIC, adoptive transfer-induced colitis consisting in infusion of naive CD4^+^ T cells in Rag2-deficient mice; CIA, Collagen-induced arthritis; CNS, central nervous system; DSS, dextran sulfate sodium; EAE, experimental autoimmune encephalomyelitis; iNOS, inducible nitric oxide synthase; LPS, lipopolysaccharide; mLN, mesenteric lymph nodes; MOG, myelin oligodendrocyte glycoprotein; TNBS, trinitrobenzene-sulfonic acid*.

In this paper, we aim to provide an overview of the available experimental evidence as well as the clinical studies that were performed in this field and that found a link between salt intake and the development of IMDs.

## The IL-23/Th17 pathway, IL-17 and Th17 cells: implication in IMDs

IMDs are complex chronic inflammatory diseases mediated by immune mechanisms involving innate immunity, as well as different T lymphocyte subsets. During the inflammatory process, naive CD4^+^ T cells differentiate into various CD4^+^ T cell subsets according to the environmental cytokine milieu. In the presence of IL-12, CD4^+^ T cells differentiate into Th1 cells typically producing IFN-γ and TNF-α and expressing the T-bet transcription factor. This lymphocyte subset has been implicated in RA, CD, psoriasis, and also MS. In contrast, Th2 cells express GATA-3 transcription factor, produce IL-4 and IL-13 and play a role in allergic diseases, parasitic infections and to a certain extent in SLE. The so-called Th17 population is characterized by its production of IL-17, IL-21, IL-22, and IL-26. IL-17 corresponds to a family of cytokines which includes six members, IL-17A to IL-17F (Kikly et al., 2006), with Th17 cells producing IL-17A and IL-17F. Th17 cells have been reported to be implicated in the natural host defense by fighting against extracellular bacteria or fungi, in granulopoiesis (Gaffen et al., 2014), neutrophil migration and activation, but also in inflammatory processes and IMDs (Miossec et al., 2009). IL-17A and IL-17F are implicated in inflammatory responses associated with autoimmune conditions, but, in general, associated with other inflammatory cytokines produced by Th17 cells, such as IL-21 (Muranski and Restifo, 2013) and IL-22 (Bettelli et al., 2006). “Typical” or “conventional” Th17 cells have been described as a main—but not the only—cellular population responsible for IL-17 production. Indeed, a large variety of innate and adaptive immune cells are responsible for IL-17A and IL-17F production, including CD8^+^ T cells, CD4^−^/CD8^−^ α/β T lymphocytes, γ/δ T cells, NK cells, NKT cells, neutrophils, innate lymphoid cells (ILC), and especially ILC3 cells, and mucosal-associated invariant T (MAIT) cells (Venken and Elewaut, 2015). The differentiation of naive CD4^+^ T cells toward the Th17 subset involves the expression of the specific retinoic acid receptor- related orphan receptor γ-T (RORγt) transcription factor (Ivanov et al., 2006). The differentiation of Th17 is also dependent on the environmental cytokine milieu that includes IL-6, TGF-β, and IL-1β. IL-23, which comprises two chains (IL-23p19 and p40), is another master cytokine involved in naive CD4^+^ T cell differentiation toward the Th17 subset (Korn et al., 2009; Gaffen et al., 2014; DuPage and Bluestone, 2016). IL-23 after interaction with its receptor sharing two chains, the IL-23R and IL-12Rβ1, favors Th17 stabilization (Langrish et al., 2005), but is also critical for maturation of pathogenic inflammatory Th17 cells by inhibiting IL-10 production (Gaffen et al., 2014). Indeed, Th17 cells represent heterogeneous Th cell subsets with pathogenic Th17 cells and regulatory anti-inflammatory Th17 cells (Gaffen et al., 2014). Moreover, Th17 cells are characterized by high plasticity and are related to both Th1 cells and Treg generated in the periphery (called peripheral Treg [pTreg]) (Korn et al., 2009). Pathogenic Th17 cells may acquire the Th1 T-bet transcription factor and the capacity to secrete IFN-γ to become highly inflammatory (Muranski and Restifo, 2013).

It is noteworthy that the IL-23/Th17 pathway is implicated in the pathophysiology of chronic IMDs in humans (Toussirot, 2012), as follows:

- RA is a chronic inflammatory joint disease with a progressive destructive course. The first report of the involvement of IL-17A in arthritis was described in 1998 with the detection of IL-17A in synovial tissues from patients with RA (Chabaud et al., 1998). In mice, injection of IL-17A causes cartilage damage (Chabaud et al., 1998). High levels of IL-17A correlate with disease severity in collagen-induced arthritis (CIA) (Nakae et al., 2003). RA synovium can produce high levels of IL-17. IL-17 is observed in low concentrations in the serum of patients with RA but is elevated in the synovial fluid (Miossec, 2009). Synovial tissues from patients with RA produce active IL-17A in addition to IL-6, TNF-α, and IL-1β. In patients with RA, expression of IL-17A in the synovium correlates with both activity and severity of the disease (Miossec, 2009).
- Psoriasis is a common chronic skin inflammatory disease characterized by keratinocyte proliferation. A heavy cellular infiltrate composed of memory T cells with high IFN-γ expression is observed in skin biopsies. It is now clear that IL-23 plays a crucial role in psoriasis: IL-23 activates macrophages and supports chronic inflammation *via* the induction of Th17 cells (Tang et al., 2012). IL-23 expression is significantly increased in the skin of patients with psoriasis, especially in psoriatic lesions compared to biopsies of normal adjacent skin (Durham et al., 2015). The importance of IL-17A in the pathogenesis of psoriasis has been well documented by genetic studies and data from experimental or animal models of psoriasis and skin tissue biopsy analysis. Genome-wide association studies have identified genetic polymorphisms that are linked to psoriasis. Indeed, the *IL-23r* gene polymorphism plays a protective role against the development of psoriasis, as well as CD (Capon et al., 2007). Moreover, Th17-associated cytokines, such as IL-22, are critical to induce the skin lesions present in psoriasis (Muranski and Restifo, 2013).
- It is believed that CD is characterized by dysregulation of Th1 cytokine production, with a role of IL-12. However, a plethora of other cytokines is found in the gut of patients with CD, including IFN-γ, as well as TNF-α, IL-1β, and IL-6. IL-23 and IL-17 are also over-expressed in the gut of patients with CD (Fujino et al., 2003). *IL-23* mRNA is highly expressed in biopsy specimens of patients with CD, and serum levels of IL-17A are increased in patients with active disease compared to patients who have inactive disease (Tang et al., 2012). An anti-IL-23 antibody targeting its specific p19 subunit is able to cure experimental colitis in mice (Elson et al., 2007).
- MS is an inflammatory disease that affects the central nervous system. Chronic inflammation in the brain results in destruction of myelin sheaths, leading to clinical manifestations. It has been demonstrated that the IL-23/Th17 pathway is involved in the animal model of MS, i.e., EAE (Cua et al., 2003). Levels of IL-17 correlate with disease severity in EAE. Monocyte-derived dendritic cells from patients with MS released more IL-23 than healthy controls (Matusevicius et al., 1999). A higher percentage of peripheral blood or cerebrospinal fluid monocytes of MS patients was positive for *IL-17* mRNA compared to cells from healthy controls (Matusevicius et al., 1999). Stimulated CD4^+^ T cells from MS patients released more IL-17A compared to T cells from healthy subjects.

## Relationships between sodium and cells of the immune system

**Figure 1:**
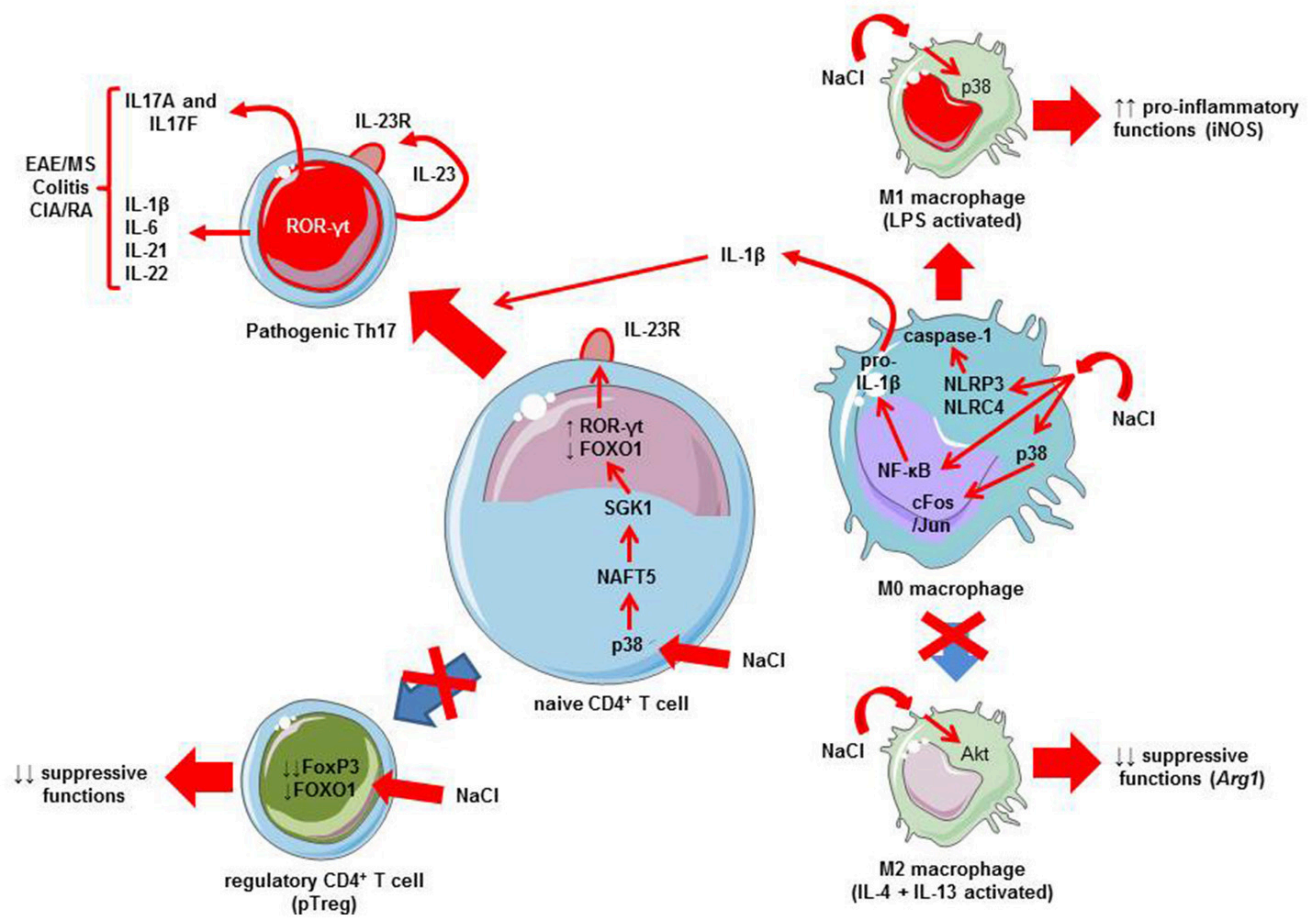
Mechanisms linking salt (NaCl) to Th17 differentiation and autoimmune diseases. A high salt intake/environment stimulates the activation of LPS-induced pro-inflammatory M1 macrophages and blunts the suppressive functions of IL-4 plus IL-13-activated M2 macrophages. A high salt intake/environment induces the activation of M0 macrophages *via* three different pathways: the p38 MAPK/cFos/Jun pathway, NF-κB activation which increases pro-inflammatory cytokine mRNAs including those coding pro-IL-1β, and finally, NLRP3/NLRC4 inflammasome which leads to caspase-1 activation responsible for pro-IL-1β cleavage into active IL-1β. IL-1β produced by macrophages exposed to a high salt environment may favor Th17 differentiation. In addition to macrophages, a high salt intake/environment directly affects CD4^+^ T cells. The activation of p38 MAPK by sodium chloride promotes the activation of nuclear factor of activated cells 5 (NFAT5), and then serum glucocorticoid kinase-1 (SGK1). SGK1 induces the phosphorylation of the transcription factor forkhead box protein O1 (FOXO1), a nuclear factor that represses the expression of the *Il23r* gene. Conversely, ROR-γt is induced leading to the transcription of the *Il23r* gene, resulting in the expression of IL23R at the membrane surface favoring the differentiation of naïve CD4^+^ T cells toward a Th17 phenotype, and thus production of IL17A and IL-17F. This favors IMD, such as experimental autoimmune encephalomyelitis (EAE), a murine model of multiple sclerosis (MS), colitis, or collagen-induced arthritis (CIA), an experimental model of rheumatoid arthritis (RA). Note that other cytokines can be released by Th17 cells, such as IL-21, IL-22, IL-1β, or IL-6. Furthermore, a high salt intake/environment inhibits the suppressive functions and the expression of FoxP3 *via* the SGK1/FOXO1 pathway.

Experimental data have reported in the nineties that mononuclear cells may release pro-inflammatory cytokines (e.g., IL-1β) when cultured in a hypertonic milieu (Shapiro and Dinarello, 1995). Later, it has been shown that sodium chloride favors pro-inflammatory M1 phenotype of macrophages (Hucke et al., 2016) and limits M2 macrophage activation (Binger et al., 2015a) (Figure 1). The pro-inflammatory effects of sodium chloride on M1 macrophages are observed in both mouse and human macrophages (Zhang et al., 2015). Several signaling pathways are triggered by elevated sodium chloride concentrations, namely: mitogen-activated protein kinases (MAPK), such as p38 kinase (Zhang et al., 2015; Hucke et al., 2016) and NF-κB (Hucke et al., 2016) (Figure 1). Macrophages may also activate the NLRP3 and NLRC4 inflammasomes in the setting of an osmotic milieu (generated for instance by high concentrations of sodium chloride) (Ip and Medzhitov, 2015). This leads to the release of IL-1β (Zhang et al., 2015). This cytokine IL-1β is critical for pathogenic human Th17 cell differentiation by inhibiting IL-10 and inducing IFN-γ (Sallusto, 2016). The direct implication of macrophages and inflammasome activation in high-salt diet-induced Th17 polarization has been demonstrated using caspase-1 deficient mice (Ip and Medzhitov, 2015). Thus, high-salt diet is responsible for innate immune cell activation that may in turn affect CD4^+^ polarization. Besides this influence on innate immune cells, it has recently been demonstrated that salt can influence adaptive immunity by favoring the Th17 pathway. Indeed, two initial studies provide strong data demonstrating the direct influence of salt in the differentiation of naive CD4^+^ T cells toward Th17 cells. Wu et al. performed a genome-wide mRNA analysis to identify the mechanisms that can explain the development of Th17 cells (Wu et al., 2013). Serum glucocorticoid kinase-1 (SGK1) was identified as a relevant candidate for IL-23R signaling and highly induced with Th17 differentiation. SGK1 expression is increased in specific conditions, including mineralocorticoid excess and hypertonicity, such as high salt exposure (Arora, 2013; Binger et al., 2015b). Indeed, SGK1 was expressed in Th17 cells and its expression strongly correlated with IL-23R signaling. This was shown by network analysis of the transcriptional changes in wild type animals and SGK1-deficient mice (Wu et al., 2013). Of interest, SGK1 was not equally expressed in other T cells subsets, especially Th1, Th2 and *in vitro* TGF-β-induced Treg (iTreg). IL-23 has the capacity to induce and maintain the expression of SGK1 in Th17 cells. Using protein-protein interaction analysis from a large database, FOXO1 was identified as a transcriptional factor that had a downstream role in the expression of SGK1. SGK1 promoted phosphorylation of FOXO1, which led to reduced FOXO1 activity and increased *IL23r* mRNA expression and induction of RORγt, a major regulator of Th17 differentiation (Wu et al., 2013). The second study by Kleinewietfeld et al. reported similar results: CD4^+^ T cells cultured in a medium with increased salt concentration regulated SGK1 expression, and thus promoted Th17 differentiation (Kleinewietfeld et al., 2013). In addition, it was shown that high salt condition activates the p38/MAPK pathway involving the nuclear factor of activated T cells, NFAT5, which in turn promotes activation of SGK1. Collectively, these data support a role for salt in SGK1 induction and the promotion of Th17 differentiation *via* signaling mechanisms involving FOXO1 and RORγt (Figure 1).

In parallel to these effects on the IL-23/Th17 pathway, salt exerts some influence on other T cell subsets, namely natural/thymic Treg (tTreg; i.e., generated in the thymus; Hernandez et al., 2015; Wu et al., 2018) and peripheral T reg (pTreg; i.e., differentiated in the periphery from naïve CD4^+^ T cells; Wu et al., 2018). Hypertonic culture medium or a high-salt diet impairs Treg function (Hernandez et al., 2015). Sodium chloride increases the release of IFN-γ by Treg thus promoting their differentiation toward a Th1 phenotype. Conversely, reducing IFN-γ may restore the suppressive activity of Treg. These effects on Treg were mediated *via* SGK1 activity. A recent paper confirms this implication of SGK1 as a negative regulator of both tTreg and pTreg (Wu et al., 2018). In fact, SGK1 represses the expression of Foxp3- the master transcription factor regulating Treg differentiation and function—via the regulation of IL-23R and reduced FOXO1 nuclear exclusion (Wu et al., 2018). The salt-sensing transcription factor SGK1 plays thus a critical role in regulating the balance of pro-inflammatory Th17 and Treg (Figure 1). High-salt diet via SGK1 activation tips the balance in favor of pro-inflammatory Th17.

Efforts have been performed to link high salt diet with modification of T cell subsets in healthy volunteers. Indeed, data are also available in human healthy subjects. A high monocyte count associated with elevated levels of circulating pro-inflammatory cytokines (e.g., IL-6, IL-17A, or IL-23) has also been reported in normal subjects who have a high salt diet (Zhou et al., 2013; Yi et al., 2015). Conversely, a reduction in salt intake is associated with a decrease in inflammatory cytokine production, such as IL-6 and IL-23 (Yi et al., 2015). A recent well-controlled study performed in 8 healthy volunteers has demonstrated the increase of circulating Th17 cells after high-salt diet ingestion (Yi et al., 2015). This confirms experimental data and the induction of potential pathogenic humans Th17.

## Influence of salt diet on IMDs

The data reported above provide a strong rationale for examining the effects of sodium chloride on the development of IMDs, especially those with a Th17 contribution. We thus focussed our review on the diseases where IL-17A has proven to be a key player, i.e., RA, psoriasis, CD, and MS. We discuss here the available experimental data together with the clinical studies that were performed on this topic. For clinical studies, we reviewed the available literature in Medline via a Pubmed search for articles published between 2013 (date of the first experimental reports; Kleinewietfeld et al., 2013; Wu et al., 2013) and 2017, as well as abstracts from the American College of Rheumatology (ACR) meetings. The MeSH terms used were “salt” OR “sodium intake” OR “sodium chloride” OR “dietary sodium intake” OR “dietary sodium” AND “autoimmune diseases” OR “rheumatoid arthritis” OR “multiple sclerosis” OR “Crohn's disease” OR “psoriasis.” To be selected for analysis, a study had to meet the following criteria: it had to be an epidemiological or pathophysiological study designed to examine the relationship between the development of RA, psoriasis, CD, or MS, and salt consumption, or to evaluate the relationship between salt diet and disease activity/severity. The Pubmed search retrieved 490 articles. After applying our selection criteria, 9 papers were selected (Table 2).

**Table 2:** Clinical studies examining the relationships between salt intake and development or activity/severity of the immune-mediated diseases.

**Author (Reference)**	**Disease**	**Number of subjects**	**Evaluation of salt intake**	**Results**
Jiang (Jiang et al., 2016)	RA	1285	Food frequency questionnaire	↑ risk of ACPA positive RA in patients with medium/high salt diet in smokers Ever smokers OR 1.3 [0.9-1.9] Heavy smokers OR 2.1 [1-1.4]
Subdström (Sundström et al., 2015)	RA	386	Food frequency questionnaire	↑ risk for the development of RA among smoking subjects in the highest tertile of salt intake OR 2.26 [1.06-4.81]
Saldago (Salgado et al., 2015)	RA	392	Validated questionnaire	↑risk of RA among never smokers with high sodium intake
Marouen (Marouen et al., 2017)	RA	24 RA and 24 controls	24 H sodium urinary excretion	Urinary sodium excretion in RA > controls. Relationship between urinary sodium and presence of erosions
McDonald (McDonald et al., 2016)	MS	170 pediatric MS	Block kids food screener	No association between sodium intake and risk of MS
Nourbakhsh (Nourbakhsh et al., 2016)	MS	174 pediatric MS	Block kids food screener	No association between sodium intake and time to relapse
Farez (Farez et al., 2015)	MS	70 adult MS	Urinary sodium excretion	Correlation between sodium intake and clinical exacerbation rate OR medium salt intake 2.75 [1.3-5.8] OR high sodium intake 3.95 [1.4-11.2]
Fitzgerald (Fitzgerald et al., 2017)	MS	465 adult CIS	24H urinary sodium excretion	No influence of salt on disease course or activity
Khalili (Khalili et al., 2016)	CD	273 CD (and 335 UC)	Food frequency questionnaire	No association between sodium intake and CD development

*RA, rheumatoid arthritis; MS, multiple sclerosis; CD, Crohn's disease; CIS, clinically isolated syndrome; UC, ulcerative colitis; OR, Odds ratio [95% confidence interval]; ACPA, anti-citrullinated protein antibodies*.

The results are discussed for each IMD, first experimental data followed by clinical studies that were found by our systematic literature review.

### Salt diet in RA

#### Experimental data (Table 1)

Two studies were performed in murine arthritis and both were available as an abstract presented at the 2014 ACR meeting (Jung et al., 2014; Sehnert et al., 2014). These two studies were performed in the CIA model which shares with human RA several clinical, histopathological, and immunological features. These latter features consists in the breach of tolerance with the implication of pathogenic T cells, as well as the production of auto-antibodies against self-antigens, such as collagen. In the first study, a low salt diet was associated with decreased joint severity compared to a high salt diet. In addition, animals with a high salt diet had elevated levels of pathogenic antibodies (IgG2a) against the autoantigen, type II collagen (Sehnert et al., 2014). The second study confirms that mice fed with a high- salt diet had more severe joint disease. Splenocytes from these animals expressed a high level of RORγt and were likely to differentiate into Th17 cells (Jung et al., 2014).

#### Clinical studies

Four published studies were found (Salgado et al., 2015; Sundström et al., 2015; Jiang et al., 2016; Marouen et al., 2017). Three examined the relationship between the intake of sodium chloride and the development of RA, and one examined the relation between sale intake and disease severity. The first study used data from a large cohort of early RA from the Netherlands (EIRA study) and analyzed the impact of sodium consumption on the development of RA among smokers according to the level of sodium intake (Jiang et al., 2016). Sodium intake was evaluated by means of a self-report questionnaire. As expected, smoking status was found to be associated with anti-citrullinated protein antibody (ACPA)-positive RA, but only in those with medium or high sodium intake [odds ratio (OR): 1.7 and 2.09 for ever-smokers and heavy-smokers, respectively]. Conversely, medium or high sodium consumption was associated with an increased risk of ACPA-positive RA among smokers only (OR 1.3 and 2.1 in ever-smokers and heavy-smokers, respectively). A Swedish nested case -control study that included 386 subjects and 1,886 matched controls examined the interaction between dietary sodium, smoking and the risk of RA (Sundström et al., 2015). Sodium intake was quantified according to a food questionnaire. Dietary habits were collected before the onset of RA. The results showed no significant association between sodium intake and the development of RA when all individuals were examined. However, there was an increased risk of RA among smokers according to the tertile of sodium consumption, with those in the highest tertile having more than a 2-fold increase in risk compared to the subjects in the lowest tertile (OR 2.26; 95%CI 1.06–4.81). Thus, these 2 studies suggest an interaction between smoking and high sodium consumption for the development of RA. A third study performed in Spain gave contradictory results (Salgado et al., 2015). In that study, daily sodium intake was estimated from a validated questionnaire. This study included 18,555 subjects and among them, 392 developed RA. The results showed that total sodium intake was significantly associated with RA (OR 1.5; 95%CI: 1.1–2.1) but never smokers with high sodium intake had a higher risk than ever smokers (*p* = 0.007). Finally in a case-control study, sodium intake as evaluated by 24 h urinary sodium excretion was found to be increased in a small cohort of patients with early RA (Marouen et al., 2017). Sodium excretion was also greater in patients with radiographic erosions compared to those without.

### Salt diet in crohn's disease

#### Experimental data (Table 1)

Normal intestinal lamina propria mononuclear cells—extracted from macroscopically and microscopically unaffected colonic samples of 9 patients undergoing resection for cancer colon- express high quantities of IL-17A, IL-23R, TNF-α, and RORγt when exposed to increasing concentrations of sodium chloride (Monteleone et al., 2017). In a mouse model of colitis, mice receiving a high salt diet developed more severe colitis that was abrogated by a pharmacologic agent that controlled p38/MAPK, and thus SGK1 (Monteleone et al., 2017). Mice that were exposed to a high salt diet had an increased frequency of IL-17A producing cells in the intestinal lamina propria as compared to mice fed with a normal diet (Wei et al., 2017). As observed in the previous study, 2,4,6-Trinitrobenzenesulfonic acid (TNBS)-induced colitis was exacerbated under a high salt diet, with a significant increase in Th17 responses in the colonic lamina propria. These results have been recently confirmed in another study by a different research group with a rapid induction of *Sgk1* mRNA (90 min after high-salt diet consumption) in the colon (Aguiar et al., 2018). Furtehrmore, *IL-10* deficient mice that were exposed to a high-salt diet developed also a more severe spontaneous colitis (Tubbs et al., 2017).

#### Clinical studies

One study examined the relationship between salt consumption and CD (Khalili et al., 2016). The subjects evaluated were women from the Nurse Health Studies (NHS and NHSII studies), which included detailed information on lifestyle and diet. The effect of salt and potassium intake was evaluated in this prospective study and incident cases of CD were recorded. Assessment of diet was performed using a 161 item semi-quantitative food frequency questionnaire. While dietary intake of potassium was found to be a protective factor for the development of CD, dietary salt intake had no influence.

### Salt diet in multiple sclerosis

#### Experimental data (Table 1)

In EAE, mice that were fed with a high salt diet developed more severe disease. High salt consumption also accelerated the onset of disease. In this setting, IL-17A expressing CD4^+^ T cells markedly infiltrated the central nervous system (Kleinewietfeld et al., 2013). Moreover, in SGK1-deficient mice, there was reduced frequency and severity of EAE (Wu et al., 2013).

#### Clinical studies

Our literature search found 4 papers analysing the effect of dietary salt in MS, namely 2 in adult patients and 2 others in pediatric subjects (Farez et al., 2015; McDonald et al., 2016; Nourbakhsh et al., 2016; Fitzgerald et al., 2017). In a multicentre case-control study performed in pediatric MS, sodium intake was evaluated using a specific pediatric food questionnaire (McDonald et al., 2016). In this study, salt intake was not related to MS: there was no difference in mean sodium intake between cases and controls. These results were confirmed in another study that evaluated dietary sodium intake using the same questionnaire (Nourbakhsh et al., 2016). Dietary sodium intake was not found to be associated with time to relapse of neurological disease. In adult patients, one study reported a significant association between sodium intake and disease activity of MS as evaluated clinically (by the expanded disability status scale-EDSS) or by central nervous system MRI (Farez et al., 2015). Sodium intake was evaluated in urine samples. There was a positive correlation between exacerbation rates and sodium excretion. Moreover, neurological exacerbation rate was 2.75 to 3.95-fold higher in patients with medium or high sodium intake compared to patients in the low intake group. These results were not confirmed by the BENEFIT study (Fitzgerald et al., 2017). This study examined the relationship between conversion from an early form of MS (i.e., clinically isolated syndrome) to MS, and urinary sodium concentration. The results showed no association between sodium intake and the conversion to MS over a 5 year follow-up period (Hazard ratio 0.91; 95%CI: 0.67–1.24). In the same way, salt intake did not correlate with disease outcome such as clinical or MRI measurements.

### Salt diet and psoriasis

We found neither experimental nor clinical data examining the relationship between salt consumption and psoriasis.

## Discussion

Sodium is an essential nutrient for the organism and is a major physiologic player. The description of its role as an environmental factor in inflammation, and especially in IMDs comes from several reports (Arora, 2013; Croxford et al., 2013; van der Meer and Netea, 2013). Indeed, strong experimental data support its implication as a driver of IL-17A production *via* IL-23R promotion. Indeed, the studies by Wu et al. and Kleinewietfeld et al. both reported that an increased salt concentration promotes Th17 differentiation by SGK1 involvement (Capon et al., 2007; Kleinewietfeld et al., 2013; Wu et al., 2013). A specific mechanism involving SGK1, FOXO1 and p38/MAPK/NAFT5 and IL-23R is thus described, providing an elegant demonstration of the relationship between salt and IL-17A production (Binger et al., 2015b). However, these results raise some questions, especially concerning the specific implication in human IL-23/Th17-mediated diseases.

One question is the consequence of a high salt diet on the blood or lymph node compartments. Indeed, besides the hypertonic and vasopressive effects on blood pressure, a high salt diet does not induce a high concentration in the blood or lymph nodes (van der Meer and Netea, 2013). Th17 cell differentiation is mainly performed in the secondary lymphoid organs, such as lymph nodes (Gaffen et al., 2014). Conversely, it may produce changes in the gut environment, and this may produce potential effects on local gut immune responses. In turn, this may represent a link between a diet factor, gut environment and flora (i.e., microbiota) and the immune system (Croxford et al., 2013). Modification of the microbiota is a well-described mechanism that has been implicated in the pathogenesis of diverse IMDs, especially CD. Of interest, a recent study reported the influence of a high salt diet on gut microbiota in mice, resulting in a depletion of *Lactobacillus murinus*. Treatment of mice with *L. murinus* prevented salt-induced aggravation of actively-induced EAE by modulating Th17 cells. A moderate high-salt intervention in a pilot study in humans reduced intestinal survival of *Lactobacillus* spp. and increased Th17 cells. These results indicate that high salt intake is connected to the gut-immune axis (Wilck et al., 2017).

Besides sodium chloride, are other salt components involved in the induction of the Th17 lymphocyte subset? For instance, potassium has been reported to be a protective cation for CD (Khalili et al., 2016). Since salt drives IL-23R expression, another question is the specific cells that could be sensitive to this environmental factor. Most experimental studies focussed the analysis on T lymphocytes, but IL-17A can be release by a wide range of other cells than Th17, including ILC3 and MAIT cells (Venken and Elewaut, 2015). These 2 cellular subsets are present in the gut, and thus, may be responsive to a salt diet. A recent study reports that ILC3 frequency is increased 2 weeks after high salt diet consumption in the mouse colon (Aguiar et al., 2018). This is an interesting track to be further explored. The experimental data reported an influence of salt in the initiating step of autoimmune diseases such as EAE or CIA, but what are the effects of salt/sodium chloride when the disease is at an established or chronic phase? Indeed, salt has a greater effect on initiation than on the maintenance of Th17 response (van der Meer and Netea, 2013).

Despite strong experimental reports, clinical studies did not report consensual results. Indeed, clinical evidence for the relationships between sodium consumption and development and/or activity/severity of IMDs is limited. Three studies reported such a link in the setting of RA (Sundström et al., 2015; Jiang et al., 2016; Sallusto, 2016; Marouen et al., 2017), and only in one study that was performed in adult patients with MS (Farez et al., 2015). One major limitation of these studies is the tool used for evaluating salt consumption, mainly a self-report questionnaire. A more accurate method involving quantitative measurements of urinary excretion was used in a limited number of studies (Farez et al., 2015; Fitzgerald et al., 2017; Marouen et al., 2017). In addition, the interaction between salt and other environmental factors (such as tobacco) was only examined in RA, giving contradictory results (Salgado et al., 2015; Sundström et al., 2015). This highlights the fact that there are indisputably multiple contributors to the development of IMDs, and they can interact with each other, as with tobacco and salt in RA. The interaction between salt and the genetic background of IMDs was not examined and it is certainly another potential avenue that warrants exploration, as it has previously been reported that there is a link between tobacco and the expression of the shared epitope for the risk of ACPA-positive RA (Klareskog et al., 2006). *SGK1* gene polymorphism is another genetic background that merits investigation, despite negative findings in RA (Jiang et al., 2016). For MS, the study by Farez et al. provided evidence that salt may worsen disease activity (Farez et al., 2015), but these results were not confirmed in the BENEFIT study (Fitzgerald et al., 2017). Moreover, epidemiological data do not support a strong influence of salt in MS occurrence. Indeed, while people from Asian countries are exposed to a high salt diet, the incidence of MS in Asian countries is among the lowest worldwide (Bach, 2018). In addition, studies examining the effect of salt in psoriasis (but also in spondyloarthritis including psoriatic arthritis), an IMD with a strong Th17/IL-17A involvement, are currently lacking. Finally, a further avenue to explore is a diet-specific intervention in these different IMDs during clinical trials. Randomized trials with a low salt diet vs. normal diet in different populations worldwide would certainly provide relevant information on the specific influence of salt in IMDs, at least in terms of disease activity. In this regard, a low salt diet intervention was performed in patients with RA or SLE, and controlled by urinary excretion measurements (Scrivo et al., 2017). In both diseases, Th17 cells decreased while there was an increase in Treg cells. These results suggest that restricted sodium dietary intake may dampen the inflammatory response in RA and SLE patients (Scrivo et al., 2017). The influence of salt diet on the response to IL-23p40 or IL-17A blocking agents (ustekinumab and secukinumab/ixekizumab, respectively) in patients with psoriasis, CD or spondyloarthritis is another relevant question to examine.

## Conclusion

The influence of the environment on the development and clinical expression of IMDs is an exciting and challenging issue. The role of sodium chloride in IMDs such as RA, CD, psoriasis and MS is a relevant question for the pathogenesis of these diseases but also for the management of patients. Current experimental data strongly support a link between salt intake and Th17 differentiation. However, the specific influence of dietary salt intake on the expression of IMDs in humans is still not demonstrated, and requires further studies, especially clinical trials with nutritional interventions aimed at comparing low vs. high salt diets. The interaction between salt and other environmental factors, as well as the genetic background, is a promising avenue for further research, in order to improve our understanding of the specific role of sodium chloride in the pathogenesis of IMDs.

## Author contributions

ET, MB, CV, and PS analyzed and discussed the literature and conceived the outline of the manuscript. ET and PS wrote the manuscript. All authors reviewed the manuscript and provided critical discussion and input.

### Conflict of interest statement

The authors declare that the research was conducted in the absence of any commercial or financial relationships that could be construed as a potential conflict of interest.
